# Metabolic syndrome among treatment‐naïve people living with and without HIV in Zambia and Zimbabwe: a cross‐sectional analysis

**DOI:** 10.1002/jia2.26047

**Published:** 2022-12-15

**Authors:** Belinda V. Chihota, Ardele Mandiriri, Tinei Shamu, Guy Muula, Hellen Nyamutowa, Charlotte Taderera, Daniel Mwamba, Roma Chilengi, Carolyn Bolton‐Moore, Samuel Bosomprah, Matthias Egger, Cleophas Chimbetete, Gilles Wandeler, for IeDEA Southern Africa

**Affiliations:** ^1^ Centre for Infectious Disease Research Lusaka Zambia; ^2^ Graduate School of Health Sciences University of Bern Bern Switzerland; ^3^ Institute of Social and Preventive Medicine University of Bern Bern Switzerland; ^4^ Newlands Clinic Harare Zimbabwe; ^5^ Department of Medicine University of Alabama at Birmingham Birmingham Alabama USA; ^6^ Department of Biostatistics School of Public Health University of Ghana Accra Ghana; ^7^ Centre for Infectious Disease Epidemiology and Research University of Cape Town Cape Town South Africa; ^8^ Population Health Sciences Bristol Medical School, University of Bristol Bristol United Kingdom; ^9^ Department of Infectious Diseases Bern University Hospital University of Bern Bern Switzerland

**Keywords:** metabolic syndrome, HIV epidemiology, Africa, women, public health, risk factors

## Abstract

**Introduction:**

Chronic viral replication has been linked to an increased risk of cardiovascular and metabolic diseases in people living with HIV (PLWH), but few studies have evaluated this association in Southern Africa. We explored the determinants of metabolic syndrome (MetS) among treatment‐naïve adults living with and without HIV in Southern Africa.

**Methods:**

Treatment‐naïve PLWH and people living without HIV (PLWOH) ≥30 years were consecutively enrolled from primary care clinics in Zambia and Zimbabwe. PLWOH were seronegative partners or persons presenting for HIV testing. We defined MetS as the presence of central obesity plus any two of the following: raised blood pressure, impaired fasting glucose, reduced high‐density lipoprotein cholesterol and raised triglycerides, as defined by the International Diabetes Federation. We used logistic regression to determine factors associated with MetS.

**Results:**

Between August 2019 and March 2022, we screened 1285 adults and enrolled 420 (47%) PLWH and 481 (53%) PLWOH. The median age was similar between PLWH and PLWOH (40 vs. 38 years, *p <* 0.24). In PLWH, the median CD4^+^ count was 228 cells/mm^3^ (IQR 108–412) and the viral load was 24,114 copies/ml (IQR 277–214,271). Central obesity was present in 365/523 (70%) females and 57/378 males (15%). MetS was diagnosed in 172/901 (19%, 95% confidence interval [CI] 17–22%), and prevalence was higher among females than males (27% vs. 9%). In multivariable analyses, HIV status was not associated with MetS (adjusted odds ratio [aOR] 1.05, 95% CI 0.74–1.51). Risk factors for MetS included age older than 50 years (aOR 2.31, 95% CI 1.49–3.59), female sex (aOR 3.47, 95% CI 2.15–5.60), highest income (aOR 2.19, 95% CI 1.39–3.44) and less than World Health Organization recommended weekly physical activity (aOR 3.35, 95% CI 1.41–7.96).

**Conclusions:**

We report a high prevalence of MetS and central obesity among females in urban Zambia and Zimbabwe. Lifestyle factors and older age appear to be the strongest predictors of MetS in our population, with no evident difference in MetS prevalence between treatment‐naïve PLWH and PLWOH.

## 1 INTRODUCTION

Chronic systemic inflammation resulting from HIV viral replication and immune dysregulation seems to increase cardiovascular and metabolic disease risk among people living with HIV (PLWH) [1]. Given the global scale‐up of antiretroviral therapy (ART) and improved life expectancy among this population [2, 3], PLWH are increasingly confronted with cardiometabolic complications. In a recent study from the Netherlands comparing people living with and without HIV, key risk factors including hypertension, dyslipidaemia, smoking and physical inactivity occurred more frequently among PLWH. The overall predicted 10‐year cardiovascular risk was higher among PLWH after adjustment for age and sex, suggesting an increased risk due to HIV infection when compared to the general population [4, 5]. Additional risk factors for metabolic complications have been attributed to certain ART regimens and lifestyle factors; however, most findings come from outside sub‐Saharan Africa (SSA) [4, 6].

Metabolic syndrome (MetS) refers to a cluster of interrelated cardiovascular disease (CVD) risk factors, including central obesity, hyperglycaemia, elevated blood pressure, hypertriglyceridemia and reduced high‐density lipoprotein (HDL) cholesterol levels [7, 8]. Although its link to mortality and morbidity has been established globally, its burden in Southern Africa remains unclear. Studies have reported MetS to be higher among PLWH on ART compared to ART‐naïve people living with and without HIV; however, estimates vary greatly by geographic region [9, 10].

Southern Africa is seeing increasing trends in obesogenic societies through urbanization and lifestyle changes posing a great need to understand the drivers of metabolic complications [11]. The dual burden with HIV, which remains highly endemic in the region, pushes the need to understand the cardiometabolic changes occurring among PLWH compared to the general population. We characterized MetS among newly diagnosed ART‐naïve people living with and without HIV seeking care at three primary care clinics in urban Zambia and Zimbabwe.

## 2 METHODS

A cross‐sectional analysis of baseline data was conducted among newly diagnosed adults living with and without HIV enrolling into a dedicated non‐communicable diseases (NCD) prospective cohort established in 2019 at three primary care clinics in Southern Africa. This study and all sites are part of the International Epidemiology Databases to Evaluate AIDS (IeDEA) collaboration in Southern Africa [12].

Participants were consecutively screened and enrolled at Kalingalinga Clinic and Matero first Level Hospital in Lusaka, Zambia and Newlands Clinic in Harare, Zimbabwe. Ethical approval was obtained from the University of Zambia Biomedical Research Ethics Committee, the National Health Research Authority Zambia and the Medical Research Council of Zimbabwe. Patients provided written informed consent to participate in the study.

### 2.1 Study participants

Eligible participants were aged 30 years or older and had a recent documented HIV test result. ART‐naïve PLWH were included if they had never taken ART or were on treatment ≤4 weeks before enrolment. PLWH were initiated on ART regimen at study enrolment as per national guidelines. People living without HIV (PLWOH) were seronegative partners or persons presenting for HIV testing. All study participants attended baseline assessments between 8 June 2019 and 10 March 2022. Participants with complete baseline cardiometabolic data were included in this analysis.

### 2.2 Study procedures

Participants underwent a full clinical examination and comprehensive laboratory testing. Blood pressure was measured using an automated cuff (OMRON Bronze upper arm blood pressure monitor BP510 [Omron® Healthcare Inc]), and the average of three measurements was calculated from readings taken 5 minutes apart. Height was measured in centimetres (cm) using a SECA 0123 stadiometer and weight in kilograms (kg) using a SECA 813 digital scale. Hip and waist circumference were measured in cm using a standard metric tape measure. Socio‐demographic variables were collected by trained research staff administering standardized questionnaires. Lifestyle data included physical activity, measured using the World Health Organization (WHO) Global Physical Activity Questionnaire (GPAQ), diet assessments were conducted using the validated WHO STEP Surveillance (STEPS) short questionnaire [13] and the Alcohol Use Disorders Identification test‐concise (AUDIT‐C) questionnaire to measure alcohol consumption [14].

### 2.3 Laboratory procedures

Blood samples for HIV viral load, CD4^+^ count and full blood count were collected. Patients were required to return to the clinic fasted for a second visit which included the collection of samples for fasting plasma glucose (FPG) and lipid panels. Total cholesterol (TC/mmol/L), HDL cholesterol (HDL‐c/mmol/l), low‐density lipoprotein (LDL‐c/mmol/L) and triglycerides (mmol/L) were measured using either the Alere Afinion ^TM^ 2 Point of Care Analyzer or the Beckman Coulter AU480 at the central laboratory. Plasma samples for blood glucose were processed at the local central laboratory using the Beckman Coulter AU480 (Zambia) and the Cobas Integra 400 plus (Zimbabwe).

### 2.4 Definitions and outcomes

The primary outcome MetS was defined using the International Diabetes Federation (IDF) 2006 guidelines as the presence of central obesity (defined as waist circumference by ethnic‐specific values) plus any two additional risk factors [15]. No ethnic‐specific cut‐offs for waist circumference are available for sub‐Saharan Africans and the European cut‐offs of males ≥94 cm and females ≥80 cm were used. Body mass index (BMI) was categorized as underweight (<18.5 kg/m^2^), normal (18.5–24.9 kg/m^2^), overweight (25.0–29.9 kg/m^2^) or obese (≥30 kg/m^2^). If BMI was ≥30 kg/m^2^, central obesity was assumed even in the presence of normal or missing waist circumference. The risk factors of MetS were defined as follows:

- Raised triglycerides ≥ 1.7 mmol/L or treatment.
- Reduced HDL cholesterol < 1.03 mmol/L in males and <1.29 mmol/L in females or treatment.
- Raised blood pressure, systolic BP ≥130 or diastolic BP ≥ 85 mmHg or treatment of previously diagnosed hypertension.
- Raised fasting glucose, FPG ≥ 5.6 mmol/L or treatment of previously diagnosed type 2 diabetes.

Physical activity was calculated as the total metabolic equivalent per minute per week (MET‐min/week) as per GPAQ protocol [16]. The WHO recommended cut‐off (≥ 600 MET‐min/week) was used to define those achieving weekly recommended activity levels. In sensitivity analyses, we estimated the prevalence and risk factors of MetS using an alternative definition. The National Cholesterol Education Program Adult Treatment Panel III (ATP III), which does not require the presence of a single factor, such as central obesity for MetS diagnosis, considers the presence of any three of the following five factors: central obesity, elevated triglycerides, elevated blood pressure, reduced HDL cholesterol and elevated FPG [7].

### 2.5 Statistical analysis

Categorical data were summarized as frequencies and proportions, while continuous variables were summarized using median and percentile values. Pearson's chi‐squared test was used to test for socio‐demographic differences between people living with and without HIV. Variables included in the logistic model were selected *a priori* and a backward selection of available candidate variables was conducted to build the full multivariable model. Results with a *p*‐value *<*0.05 were considered statistically significant. All analyses were performed using STATA software, version 16.0/IC (Stata Corporation, College Station, TX).

## 3 RESULTS

### 3.1 Patient characteristics

**Figure 1:**
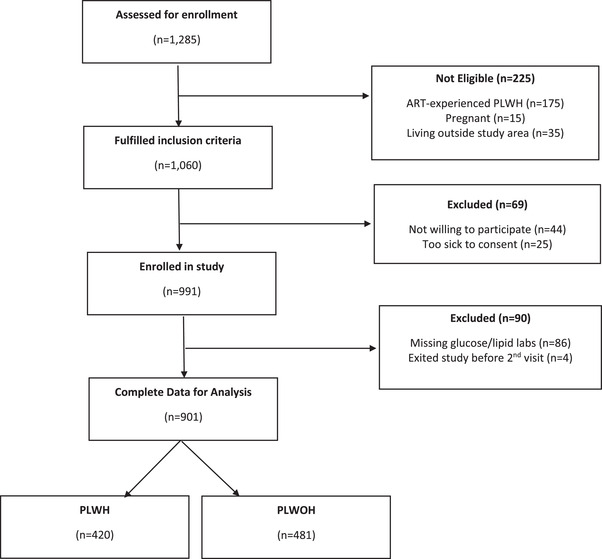
Flow chart of included participants.

Among 991 participants enrolled in the cohort, 901 (91%) had complete data for this analysis, including 420 (47%) ART‐naïve PLWH and 481 (53%) PLWOH (Table 1). Patients were excluded from this analysis if they did not have fasting glucose or lipid results (9%) or died/transferred out of the study before the second visit (1%) (Figure 1). The median age was similar among PLWH and PLWOH (40% vs. 38%*, p =* 0.24) but the proportion of females was higher in the PLWOH group (63% vs. 52%, *p <* 0.001). PLWH were more likely to be employed (73% vs. 65%, *p =* 0.02) and have a college/university degree (33% vs. 22%, *p <* 0.001). There were no differences in income levels between the two groups (*p <* 0.40). The majority of the participants resided in high‐density residential areas, with a higher proportion of PLWOH individuals dwelling in peri‐urban areas (35% vs. 14%, *p <* 0.001). Among PLWH, the median CD4^+^ cell count was 228 cells/mm^3^ (IQR: 108–412), the median HIV viral load was 24,114 copies/ml (IQR: 277–214,271) and 87% initiated an ART regimen including tenofovir disoproxil fumarate, lamivudine and dolutegravir (TDF/3TC/DTG) at enrolment.

**Table 1:** Baseline characteristics by HIV status

	PLWH	PLWOH	
Socio‐demographic characteristics	(*n* = 420)	(*n* = 481)	*p‐*value
Age years, median (IQR)	40 (34–45)	38 (32–46)	0.24
Sex, *n* (%)			**<0.01**
Female	218 (52)	305 (63)	
Male	202 (48)	176 (37)	
Country, *n* (%)			0.77
Zambia	219 (52)	246 (51)	
Zimbabwe	201 (48)	235 (49)	
Marital status, *n* (%)			**<0.01**
Married	229 (55)	329 (68)	
Divorced/separated	94 (22)	60 (12)	
Widowed	44 (10)	41 (9)	
Single	53 (13)	51 (11)	
Highest level of education			**<0.01**
Primary school	64 (15)	109 (23)	
Secondary school	206 (49)	250 (52)	
Tertiary (college/university)	138 (33)	105 (22)	
No formal education	12 (3)	17 (4)	
Residential area			**<0.01**
Peri‐urban	58 (14)	166 (35)	
Low/medium density	111 (26)	84 (17)	
High density	241 (57)	216 (45)	
Rural/informal settlement	10 (2)	15 (3)	
Monthly household income, *n* (%)			0.40
Lowest	116 (28)	153 (32)	
Middle	224 (53)	241 (50)	
Highest	79 (19)	87 (18)	
Employment, *n* (%)			**0** **.02**
Employed/self‐employed	304 (73)	314 (66)	
Unemployed	116 (27)	166 (34)	
HIV clinical characteristics			
Baseline CD4 count, cells/mm^3^, median (IQR)	228 (108–412)	–	–
Baseline HIV viral load, copies/ml, median (IQR)	24,114 (277–214,271)	–	–
WHO Clinical Staging		–	–
Stage I	330 (79)		
Stage II	36 (9)		
Stage III	34 (7)		
Stage IV	20 (5)		

Abbreviations: PLWH, people living with HIV; PLWOH, people living without HIV.

Bold means statistical significance *p* ≤ 0.5.

### 3.2 Prevalence of MetS and individual components

**Figure 2:**
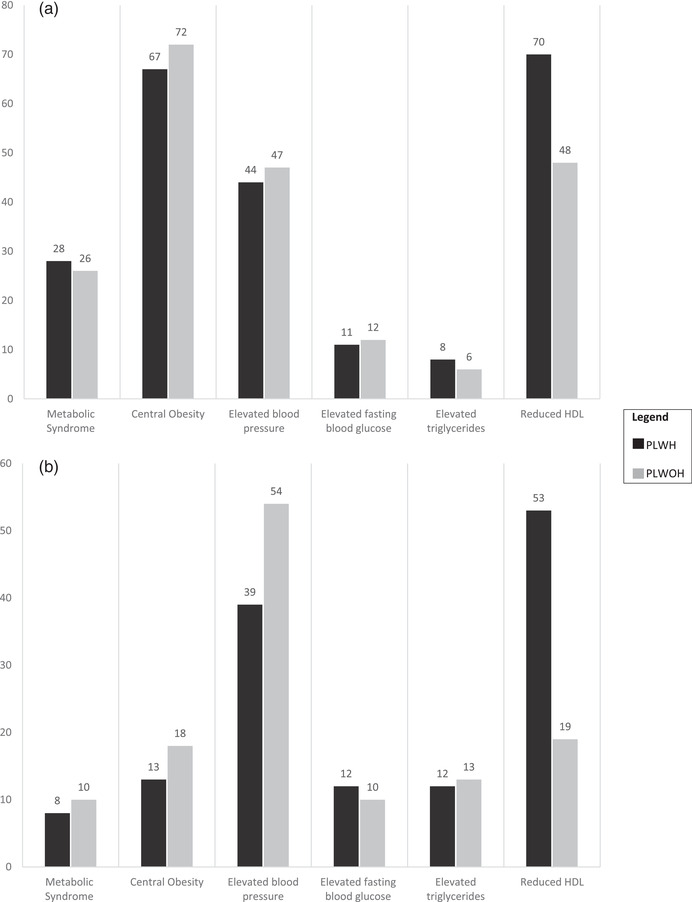
Prevalence of metabolic syndrome and risk factors by HIV status among females (a) and males (b), according to the International Diabetes Federation definition.

The overall prevalence of MetS using the IDF criteria was 19% (95% CI 17–22), with estimates reaching 22% (95% CI 18–26) in Zimbabwe and 16% (95% CI 13–20) in Zambia. Females were more likely to have MetS when compared to males irrespective of HIV status (27% vs. 9%). Figure 2 shows the proportion of females (Figure 2a) and males (Figure 2b) with each MetS component. When ATP III criteria were used to define MetS, results were very similar: 28% of females and 12% of males had MetS (Supplement 1 and Figure S4). Central obesity was strikingly higher among females regardless of HIV status (70% vs. 15%). Elevated blood pressure appeared higher among PLWOH irrespective of sex (50% vs. 42%), whereas elevated fasting blood glucose and triglycerides were comparable across all groups (Figure 1 and Table 2). The proportion with reduced HDL was higher among PLWH irrespective of sex (63% vs. 37%).

**Table 2:** Metabolic syndrome and CVD risk factors by sex and HIV status

	Males		Females	
	PLWH	PLWOH	PLWH	PLWOH
Variable	(*n* = 202)	(*n* = 176)	(*n* = 218)	(*n* = 305)
Metabolic syndrome, *n* (%)	16 (8)	17 (10)	60 (28)	79 (26)
Components of MetS criteria, *n* (%)				
Central obesity	26 (12)	31 (18)	146 (67)	219 (72)
Elevated blood pressure	79 (39)	95 (54)	95 (44)	143 (47)
Low HDL‐C	108 (53)	33 (19)	152 (70)	146 (48)
Elevated triglycerides	25 (12)	22 (13)	17 (8)	18 (6)
Elevated fasting plasma glucose	25 (12)	18 (10)	25 (11)	37 (12)
Body mass index categories (kg/m^2^) *n* (%)				
Underweight	32 (16)	15 (9)	24 (11)	10 (3)
Normal	127 (63)	121 (69)	94 (43)	112 (37)
Overweight	38 (19)	32 (18)	58 (27)	83 (27)
Obese	5 (2)	8 (5)	42 (19)	99 (32)
History of cardiovascular disease risk factors and treatment, *n* (%)				
Prior history or treatment of hypertension	12 (6)	16 (9)	32 (15)	74 (24)
Prior history or treatment of diabetes	2 (1)	1(1)	5 (2)	6 (2)
History of stroke	2 (1)	0	2 (1)	5 (2)
Current smoker, *n* (%)	49 (24)	55 (31)	2 (1)	5 (2)
Unhealthy alcohol consumption, *n* (%)	113 (56)	98 (56)	43 (20)	70 (23)
Achieving recommended physical activity ≥600 MET minutes/week, *n* (%)	193 (96)	171 (97)	211 (97)	298 (98)
Less than five servings of fruits and/or vegetables per day, *n* (%)	140 (69)	136 (77)	173 (79)	247 (81)

Abbreviations: PLWH, people living with HIV; PLWOH, people living without HIV.

### 3.3 CVD risk, behavioural and lifestyle factors

Females were more likely to be overweight/obese than males, irrespective of HIV status (54% vs. 22%) (Table 2). Overall, nine participants (1%) reported a prior history of stroke before enrolment. Previous hypertension diagnosis or treatment was highest among females living with HIV (24%) and lowest among males living with HIV (6%). Smoking and unhealthy alcohol consumption were more common in males and recommended physical activity per week was comparable across all groups. Females were more likely to have less than recommended fruit and/or vegetable daily consumption when compared to males (81% vs. 73%).

### 3.4 Factors associated with MetS

**Figure 3:**
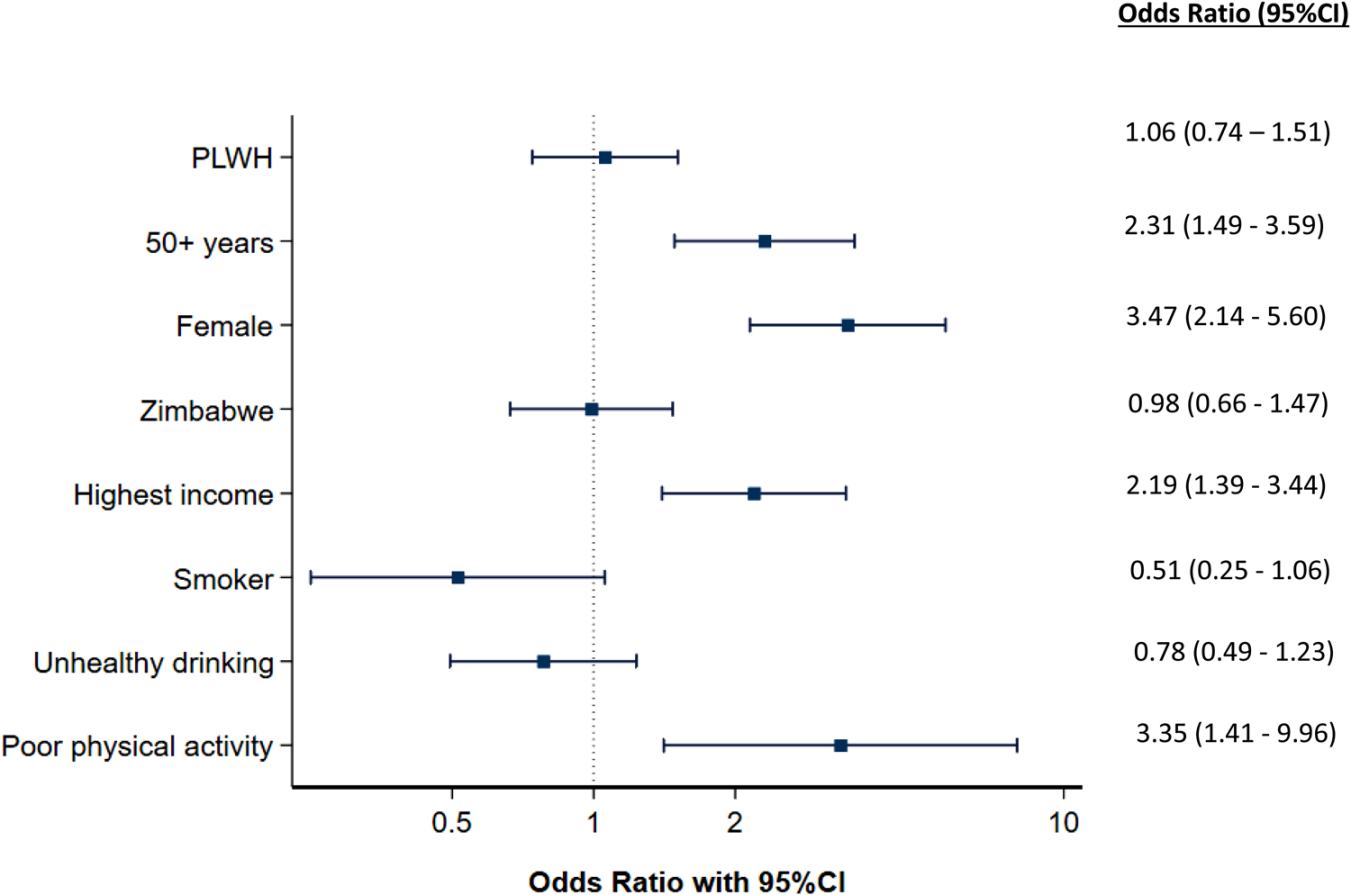
Forest plot of factors associated with metabolic syndrome according to the IDF definition (multivariable analysis).

In multivariable analyses, HIV status was not associated with MetS (adjusted odds ratio [aOR] 1.06, 95% CI 0.74–1.51 [Figure 3 and Supplement 3]). However, age older than 50 years (2.31, 95% CI 1.49–3.59]), female sex (aOR 3.47, 95% CI 2.14–5.60), high income (aOR 2.19, 95% CI 1.39–3.44) and less than WHO recommended weekly physical activity (aOR 3.35, 95% CI 1.41–9.96) were strongly associated with this outcome. When the ATP III definition was used, the same variables remained significantly associated with MetS, with similar point estimates (Supplement 2 and Figure S5).

## 4 DISCUSSION

In urban Zambia and Zimbabwe, we found a high prevalence of MetS among females, irrespective of HIV status. The presence of MetS was strongly associated with older age (≥50 years), high income and poor physical activity. More than two‐thirds of female participants had central obesity, which is a required component of the MetS IDF definition, and one‐half had an elevated BMI. With the ongoing dual HIV/NCD disease epidemics in SSA, our results highlight the need for integration of routine CVD risk factor screening and management irrespective of HIV status across primary care settings in Southern Africa.

Although estimates of MetS from SSA vary greatly, our overall prevalence of 19% is comparable to findings from Nigeria and Kenya where a prevalence of 16–17% was reported among PLWH [17, 18]. Whereas one Kenyan cohort found a higher prevalence of MetS among adults living without HIV compared to those living with HIV (12% vs. 6%), we did not find a significant difference between people living with and without HIV [19]. Although cohorts from high‐income countries have shown an increased risk of MetS among ART‐experienced PLWH, few data are available from ART‐naïve participants. Results from studies of ART‐experienced African cohorts are heterogeneous [19], whereas the prevalence of MetS was 26% in a cohort of ART‐experienced adults in Zambia [20], a cohort in Kenya found a slightly higher prevalence of MetS in the ART‐naïve population compared to ART‐experienced PLWH [17]. Future research will have to look into the impact of HIV viral replication and ART regimens on MetS development.

Sex was an important confounder in the analysis of the potential association between HIV infection and MetS [21]. Females were over three times more likely to have MetS than males in our study, which aligns with results from previous studies among PLWH on ART in the region. In a recent study of 1166 individuals on ART in Lesotho, 22% of females versus 6% of males had MetS, whereas among South African females on ART, the prevalence of MetS was 18% [18, 22, 23]. Although most available data from Southern Africa are from cohorts of PLWH on ART, our results confirm similar trends among PLWOH in urban areas. Given the high risk of MetS found among women, our results support the need to report research findings stratified by sex in order to help guide policy and recommendations.

The consensus on which definition of MetS to use remains unclear. The IDF must include the presence of central obesity, whereas the National Cholesterol Education Program Adult Treatment Panel III guidelines (ATP III) require the presence of any three out of five risk factors [24, 25]. The IDF definition may be underestimating MetS among our male population since less than 10% have central obesity, excluding those who may have three risk factors in the absence of central obesity. Although the estimates in our cohort were similar across MetS definitions, the choice of definitions has been shown to produce varying estimates even within the same population. Findings from Ethiopia found a MetS prevalence of 16% in females using the ATP III and 24% using the IDF criteria [26], highlighting the need for a standardized definition for comparisons to be made across studies.

### 4.1 Limitations

As our study was conducted in a largely urban population, our estimates may not be generalizable to the full population of these two Southern African settings. The lack of ethnic‐specific cut‐offs for central obesity may have led to an overestimation of the burden of MetS in our population. Previous studies have reported bias in BMI and waist circumference measures as they were originally derived from European populations and do not take into account ethnic or genetic differences in adiposity [27]. The lack of clinically relevant cut‐offs specific to our context makes it challenging to determine the impact of high obesity and MetS on long‐term cardiovascular and clinical outcomes in our population. Similarly, the measurement of lifestyle risk factors, such as diet and physical activity, needs further evaluation in our context. Although associations were shown between poor physical activity and MetS, the majority of our cohort were active and reaching the recommended activity levels, but still having high BMIs. The association of highest income has been reported elsewhere; however, studies from rural cohorts have equally shown MetS to be present and the addition of a rural population would have supported our findings on socio‐economic status. The significant decrease in HDL‐c among PLWH has been well documented among persons with advanced HIV disease and may have had an impact on our estimation of the prevalence of MetS in this group [28]. Furthermore, the presence of severe weight loss in some participants with advanced HIV may have led to the underestimation of MetS.

## 5 CONCLUSIONS

We report a high prevalence of MetS and central obesity among females irrespective of HIV status in an urban population in Zambia and Zimbabwe. The rollout of DTG and tenofovir alafenamide, which have been linked to weight increases and worsening lipid profiles among PLWH, could further exacerbate the burden of metabolic complications in the region [29, 30, 31]. As approximately 90% of PLWH in our study initiated a DTG‐containing regimen, the prospective monitoring of metabolic parameters in our study will improve our knowledge on the impact of contemporary ART on associated comorbidities.

## COMPETING INTERESTS

The authors declare that they have no known competing financial interests or personal relationships that could have appeared to influence the work reported in this paper.

## AUTHORS’ CONTRIBUTIONS

BVC and GW designed the study, analysed the data and wrote the first draft of the manuscript. BVC, AM, TS, GM, HN and CT collected data. CBM and CC supported study implementation at their respective sites. All authors contributed to interpretation of data, critically reviewed the manuscript and agreed on its final version.

## FUNDING

This work was funded by the National Institute of Allergy and Infectious Disease (grant number U01AI069924). GW was supported by a Professorship from the Swiss National Science Foundation (SNSF; grant number PP00P3_176944). ME was supported by special project funding (grant number 189498) from the Swiss National Science Foundation.

## Supporting information


**Supplement 1**: MetS prevalence and components using ATP III definition.
**Supplement 2**: Factors associated with Metabolic Syndrome using ATP III definition.
**Supplement 3**: Factors associated with Metabolic Syndrome using IDF definition.

## Data Availability

The data that support this study are not publicly available due to privacy and ethical restrictions. However, the data are available on request from the IeDEA‐Southern Africa consortium, and the proposed use of the data must be based on a concept note that is approved by the regional steering group.

## References

[1] Adelzon P , Falcao M , Pacheco A . Metabolic syndrome in HIV‐infected individuals: underlying mechanisms and epidemiological aspects. AIDS Res Ther. 2013; 10:32. 24330597 10.1186/1742-6405-10-32 PMC3874610

[2] Ministry of Health, Zambia . Zambia consolidated guidelines for treatment and prevention of HIV infection [Internet]. 2020 [cited 2020 Feb 18]. Available from: https://www.moh.gov.zm/wp‐content/uploads/filebase/Zambia‐Consolidated‐Guidelines‐for‐Treatment‐and‐Prevention‐of‐HIV‐Infection‐2020.pdf

[3] World Health Organization . Consolidated guidelines on HIV prevention, testimg, treatment, service delivery and monitoring: recommendations for a public health approach [Internet]. 2021 [cited 2022 Feb 18]. Available from: https://www.who.int/publications/i/item/9789240031593 34370423

[4] Schouten J , Wit F , Stolte I , Kootstra N , van der Valk M , Geerlings S , et al. Cross‐sectional comparison of the prevalence of age‐associated comorbidities and their risk factors between HIV‐infected and uninfected individuals: the AGEhIV cohort study. Clin Infect Dis. 2014; 59(12):1781–97. 10.1093/cid/ciu701 25182245

[5] van Zoest R , van der Valk M , Wit F , Vaartjes I , Kooij K , Hovius J , et al. Suboptimal primary and secondary cardiovascular disease prevention in HIV‐positive individuals on antiretroviral therapy. Eur J Prev Cardiol. 24(12):1297–307. 10.1177/2047487317714350 PMC5548068 28578613

[6] Vachiat A , McCutcheon K , Tsabedze N , Zachariah D , Manga P . HIV and ischemic heart disease. J Am Coll Cardiol. 2016; 69(1):73–82. 10.1016/j.jacc.2016.09.979 28057253

[7] Alberti K , Eckel R , Grundy S , Zimmet P , Cleeman J , Donato K , et al. Harmonizing the metabolic syndrome. A joint interim statement of the International Diabetes Federation Task Force on Epidemiology and Prevention; National Heart, Lung, and Blood Institute; American Heart Association; World Heart Federation; International Atherosclerosis Society; and International Association for the Study of Obesity. Circulation. 2009; 120(16):1640–5. 19805654 10.1161/CIRCULATIONAHA.109.192644

[8] Saklayen M . The global epidemic of the metabolic syndrome. Curr Hypertens Rep. 2018; 20(2):12. 29480368 10.1007/s11906-018-0812-z PMC5866840

[9] Sobieszcyk M , Werner L , Mlisana K , Naicker N , Feinstein A , Gray C , et al. Metabolic syndrome after HIV acquisition in South African women. J Acquir Immune Defic Syndr. 2016; 73(4):438–45. 27391387 10.1097/QAI.0000000000001123

[10] Todowede O , Mianda S , Sartorius B . Prevalence of metabolic syndrome among HIV‐positive and HIV‐negative populations in sub Saharan Africa—a systematic review and meta‐analysis. Syst Rev. 2019;8(1):4. 30606249 10.1186/s13643-018-0927-y PMC6317235

[11] Nnyepi M , Gwisai N , Lekgoa M , Seru T . Evidence of nutrition transition in Southern Africa. Proc Nutr Soc. 2015; 74(4):478–86. 25686639 10.1017/S0029665115000051

[12] Chammartin F , Ostinelli CH , Anastos K , Jaquet A , Brazier E , Brown S , et al. International Epidemiology Databases to Evaluate AIDS (IeDEA) in sub‐Saharan Africa, 2012–2019. BMJ Open. 2020; 10(5):e035246. 10.1136/bmjopen-2019-035246 PMC7232622 32414825

[13] World Health Organization . WHO STEPS Surveillance Manual: the WHO STEPwise appraoch to chronic disease risk factor surveillance. Geneva: World Health Organization; 2005.

[14] Bradley K , DeBenedetti A , Volk R , Williams E , Frank D , Kivlahan D . AUDIT‐C as a brief screen for alcohol misuse in primary care. Alcohol Clin Exp Res. 2007; 31(7):1208–17. 17451397 10.1111/j.1530-0277.2007.00403.x

[15] International Diabetes Federation . The IDF consensus worldwide definition of the metabolic syndrome [Internet]. 2006 [cited 2022 Feb 14]. Available from: https://www.idf.org/e‐library/consensus‐statements/60‐idfconsensus‐worldwide‐definitionof‐the‐metabolic‐syndrome.html

[16] World Health Organization . Global Physical Activity Questionnaire (GPAQ) analysis guide. Geneva: World Health Organization; 2021.

[17] Osoti A , Temu T , Kirui N , Ngetich E , Kamano J , Page S , et al. Metabolic syndrome among antiretroviral therapy‐naive versus experienced HIV‐infected patients without preexisting cardiometabolic disorders in Western Kenya. AIDS Patient Care STDs. 2018; 32(6):215–22. 29851503 10.1089/apc.2018.0052 PMC5982154

[18] Ayodele O , Akinboro A , Akinyemi S , Adepeju A , Akinremi O , Alao C , et al. Prevalence and clinical correlates of metabolic syndrome in Nigerians living with human immunodeficiency virus/acquired immunodeficiency syndrome. Metab Syndr Relat Disord. 2012; 10(5):373–9. 22799758 10.1089/met.2012.0050

[19] Masyuko S , Page S , Kinuthia J , Polyak S , Osoti A , Otieno FC , et al. Metabolic syndrome and 10‐year cardiovascular risk among HIV‐positive and HIV‐negative adults. Medicine. 2020;99(27):e20845. 32629671 10.1097/MD.0000000000020845 PMC7337552

[20] Hamooya B , Mulenga L , Masenga S , Fwemba I , Chirwa L , Siwinga M , et al. Metabolic syndrome in Zambian adults with human immunodeficiency virus on antiretroviral therapy: prevalence and associated factors. Medicine. 2021;100(14):e25236. 33832083 10.1097/MD.0000000000025236 PMC8036111

[21] Faulkner J , de Chantemele E . Sex hormones, aging and cardiometabolic syndrome. Biol Sex Differ. 2019; 2019(10):30. 10.1186/s13293-019-0246-6 PMC6604485 31262349

[22] Labhardt N , Muller U , Ringera I , Ehmer J , Motlatsi M , Pfieiffer K , et al. Metabolic syndrome in patients on first‐line antiretroviral therapy containing zidovudine or tenofovir in rural Lesotho, Southern Africa. Trop Med Int Health. 22(6):725–33. 28342180 10.1111/tmi.12872

[23] Hanley S , Moodley D , Naidoo M . Obesity in young South African women living with HIV: a cross‐sectional analysis of risk factors for cardiovascular disease. PLoS One. 2021;16(11):e0255652. 34780476 10.1371/journal.pone.0255652 PMC8592426

[24] Huang P . A comprehensive definition for metabolic syndrome. Dis Model Mech. 2009; 2(5–6):231–7. 19407331 10.1242/dmm.001180 PMC2675814

[25] Grundy S , Stone N , Bailey A , Beam C , Birtcher K . 2018 AHA/ACC/AACVPR/AAPA/ABC/ACPM/ADA/AGS/APhA/ASPC/NLA/PCNA Guideline on the Management of Blood Cholesterol: a report of the American College of Cardiology/American Heart Association Task Force on Clinical Practice Guidelines. Circulation. 2019; 139(25):1082–143. 10.1161/CIR.0000000000000625 PMC7403606 30586774

[26] Tran A , Gelaye B , Girma B , Lemma S , Berhane Y , Bekele T , et al. Prevalence of metabolic syndrome among working adults in Ethiopia. Int J Hypertens. 2011; 2011:8. 10.4061/2011/193719 PMC3124293 21747973

[27] Yaghootkar H , Whitcher B , Thomas B . Ethnic differences in adiposity and diabetes risk – insights from genetic studies. J Intern Med. 2020; 288(3):271–83. 32367627 10.1111/joim.13082

[28] Zangerle R , Sarcletti M , Gallati H , Reibnegger G , Wachter H , Fuchs D . Decreased plasma concentrations of HDL cholesterol in HIV‐infected individuals are associated with immune activation. J Acquir Immune Defic Syndr. 1994; 7(11):1149–56. 7932082

[29] Mallon P , Brunet L , Fusco J , Prajapati G , Beyer A , Fusco G , et al. Lipid changes after switch from TDF to TAF in the OPERA Cohort: LDL cholesterol and triglycerides. Open Forum Infect Dis. 2021; 9(1):ofab621. 35028335 10.1093/ofid/ofab621 PMC8753026

[30] Venter W , Sokhela S , Simmons B , Moorehouse M , Fairlie L , Mashabane N , et al. Dolutegravir with emtricitabine and tenofovir alafenamide or tenofovir disoproxil fumarate versus efavirenz, emtricitabine, and tenofovir disoproxil fumarate for initial treatment of HIV‐1 infection (ADVANCE): week 96 results from a randomised, phase 3, non‐inferiority trial. Lancet HIV. 2020; 7(10):e666–76. 33010240 10.1016/S2352-3018(20)30241-1

[31] Surial B , Mugglin C , Calmy A , Cavassini M , Gunthard H , Stockle M , et al. Weight and metabolic changes after switching from tenofovir disoproxil fumarate to tenofovir alafenamide in people living with HIV: a cohort study. Ann Intern Med. 2021; 174(6):758–67. 33721521 10.7326/M20-4853

