# Supplementary material for: Metabolic syndrome among treatment‐naïve people living with and without HIV in Zambia and Zimbabwe: a cross‐sectional analysis
Source: J Int AIDS Soc. 2022 Dec 15;25(12):e26047. doi: 10.1002/jia2.26047 (PMC9755006; doi:10.1002/jia2.26047)
Supplement: Supplementary file 1 — Supplement 1: MetS prevalence and components using ATP III definition. Supplement 2: Factors associated with Metabolic Syndrome using ATP III definition. Supplement 3: Factors associated with Metabolic Syndrome using IDF definition. [file JIA2-25-e26047-s001.docx]

**Supplementary Material**

**S1: MetS prevalence and components using ATP III definition**

**Figure 4a. Females**

**Legend**

PLWH

PLWOH

**Figure 4b. Males**

**Figure 4. ATP III Definition. a**) Prevalence of metabolic syndrome and risk factors by HIV status among Women **b)** Prevalence of metabolic syndrome and risk factors by HIV status among Men

**S2. Factors associated with Metabolic Syndrome using ATP III definition**

|  | n/N | Univariable Analysis  OR (95% CI) *p* | | Multivariable Analysis*  OR (95% CI) *p* | |
| --- | --- | --- | --- | --- | --- |
| HIV Status  Negative  Positive | 101/481  92/420 | Ref  1.06 (0.77-1.45) | *-*  *0.74* | Ref  1.23 (0.88 – 1.72) | *-*  *0.23* |
| Age group, years  30-49  ≥50 | 147/761  46/140 | Ref  2.04 (1.38-3.04) | *-*  ***<0.01*** | Ref  2.10 (1.37 – 3.21) | *-*  ***<0.01*** |
| Sex  Male  Female | 47/378  146/523 | Ref  2.73 (1.90-3.91) | *-*  ***<0.01*** | Ref  2.27 (1.48- 3.46) | *-*  ***<0.01*** |
| Country  Zambia  Zimbabwe | 85/465  108/436 | Ref  1.47 (1.07 – 2.03) | *-*  ***0.02*** | Ref  1.01 (0.69 – 1.48) | **-**  *0.93* |
| Monthly income  Lowest/Middle  Highest | 144/734  49/166 | Ref  1.72 (1.17 – 2.51) | *-*  ***<0.01*** | Ref  1.84 (1.44 -3.60) | ***-***  ***<0.01*** |
| Smoker  Never  Current/Past | 180/715  13/186 | Ref  0.22 (0.12 – 0.40) | *-*  ***<0.01*** | Ref  0.56 (0.27-0.79) | ***-***  *0.13* |
| Alcohol consumption  Abstainer/Moderate  Hazardous | 150/577  43/324 | Ref  0.44 (0.30 – 0.63) | **-**  ***<0.01*** | Ref  0.77 (0.48 – 1.22) | **-**  *0.26* |
| Physical Activity  Recommended  Less than recommended | 181/83  12/28 | Ref  2.87 (1.33 – 6.17) | *-*  ***<0.01*** | Ref  3.55 (1.48 – 8.54) | **-**  ***<0.01*** |

*Complete case analysis n= 900

**Figure 5. Forest plot of factors associated with metabolic syndrome (ATP III definition) (multivariable analysis)**


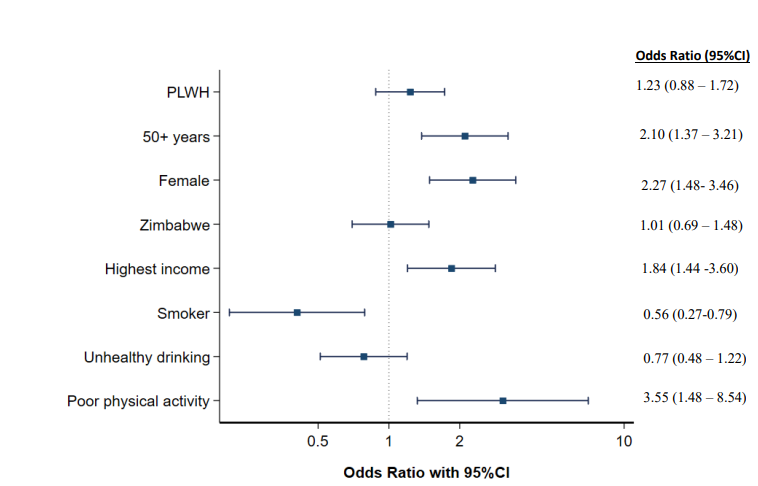


**S3. Factors associated with Metabolic Syndrome using IDF definition**

|  | n/N | Univariable Analysis  OR (95% CI) *p* | | Multivariable Analysis*  OR (95% CI) *p* | |
| --- | --- | --- | --- | --- | --- |
| HIV Status  Negative  Positive | 96/481  76/420 | Ref  0.89 (0.63 - 1.24) | *-*  *0.48* | Ref  1.06 (0.74 - 1.51) | *-*  *0.76* |
| Age group, years  30-49  ≥50 | 129/761  43/140 | Ref  2.17 (1.45 - 3.26) | *-*  ***<0.01*** | Ref  2.31 (1.49 - 3.59) | *-*  ***<0.01*** |
| Sex  Male  Female | 33/378  139/523 | Ref  3.78 (2.52 - 5.68) | *-*  ***<0.01*** | Ref  3.47 (2.14 - 5.60) | *-*  ***<0.01*** |
| Country  Zambia  Zimbabwe | 75/465  97/436 | Ref  1.49 (1.06 - 2.08) | *-*  ***0.02*** | Ref  0.98 (0.66 - 1.47) | **-**  *0.96* |
| Monthly income  Lowest/Middle  Highest | 126/734  46/166 | Ref  1.85 (1.25 - 2.73) | *-*  ***<0.01*** | Ref  2.19 (1.39 - 3.44) | ***-***  ***<0.01*** |
| Smoker  Never  Current/Past | 161/715  11/186 | Ref  0.22 (0.11 - 0.41) | *-*  ***<0.01*** | Ref  0.51 (0.25 - 1.06) | ***-***  *0.07* |
| Alcohol consumption  Abstainer/Moderate  Hazardous | 136/577  36/324 | Ref  0.40 (0.27 - 0.60) | **-**  ***<0.01*** | Ref  0.78 (0.49 - 1.23) | **-**  *0.29* |
| Physical Activity  Recommended  Less than recommended | 161/873  11/28 | Ref  2.86 (1.32 - 6.23) | *-*  ***<0.01*** | Ref  3.35 (1.41 - 9.96) | **-**  ***<0.01*** |

* Complete case analysis n= 900
